# Inadvertent catheter misplacement into the subclavian artery during ultrasound-guided internal jugular venous catheterization: a case report

**DOI:** 10.1186/s40981-023-00649-1

**Published:** 2023-09-06

**Authors:** Tomoki Kohyama, Keisuke Fujimaki, Hiroki Sasamori, Joho Tokumine, Kiyoshi Moriyama, Tomoko Yorozu

**Affiliations:** 1 Department of Anesthesia, National Hospital Organization Disaster Medical Center, 3256 Midoricho, Tachikawa, Tokyo, 190-0014 Japan; 2 Department of Cardiovascular Surgery, Kyorin University School of Medicine, 6-20-2 Shinkawa, Mitaka, Tokyo, 181-8611 Japan; 3 Department of Neurosurgery, Hayama Heart Center, 1898-1 Shimoyamaguchi, Hayama, Miura, Kanagawa, 240-0116 Japan; 4 Department of Anesthesiology, Kyorin University School of Medicine, 6-20-2 Shinkawa, Mitaka, Tokyo, 181-8611 Japan

**Keywords:** Ultrasound-guided internal jugular venous catheterization, Mechanical complication, Guidewire, Low approach

## Abstract

**Background:**

Ultrasound-guided central venous catheterization has become a standard procedure. However, mechanical complications are still reported.

**Case presentation:**

An 85-year-old woman presented with coagulopathic bladder tamponade. Ultrasound-guided right internal jugular venous catheterization was planned because of difficult peripheral venous access. A guidewire was advanced through a needle inserted at the midpoint of the right carotid triangle. The guidewire was identified in the short axis, but not in the long-axis ultrasound view, leading to inadvertent insertion of the catheter into the right subclavian artery through the internal jugular vein. Stent graft insertion was performed for perforation closure. The patient exhibited no symptoms of cerebral ischemia following stent graft insertion.

**Discussion:**

This case demonstrated that the needle-sticking site should not be placed close to the clavicle for ultrasound-guided internal jugular venous catheterization, as it may not confirm the position of guidewire in the long-axis ultrasound view.

## Background

Ultrasound guidance is a standard technique for central venous catheter placement. Real-time ultrasound guidance increases success rates [1]. However, mechanical complications can occur even with ultrasound guidance [2]. We experienced a case of inadvertent insertion of a catheter into the right subclavian artery during ultrasound-guided internal jugular vein catheterization (US-IJV). This case suggests the adverse effects of the latter, in which it is difficult to confirm the guidewire position via long-axis ultrasound imaging.

## Case presentation

An 85-year-old woman (height: 160 cm, body weight: 48.4 kg, BMI: 19) was admitted to our hospital for persistent gross hematuria. She had right hemiplegia caused by cardiogenic stroke due to chronic atrial fibrillation, which was treated using clopidogrel 75 mg/day and dabigatran 110 mg/day. The hematuria was presumed to be due to bladder hemorrhage as a side effect of clopidogrel and dabigatran. Therefore, the use of these drugs was discontinued. Seven days later, the hematuria increased, and urinary retention occurred, and she was diagnosed coagulopathic bladder tamponade. Emergent intravesical thrombectomy was scheduled for the removal of the bladder coagula. Prior to surgery, the emergency physician attempted to obtain peripheral venous access for blood transfusion for 2 h but was unsuccessful. During this time, the patient’s blood pressure started to gradually drop, and she was deemed to be at risk of going into hemorrhagic shock.

**Fig. 1:**
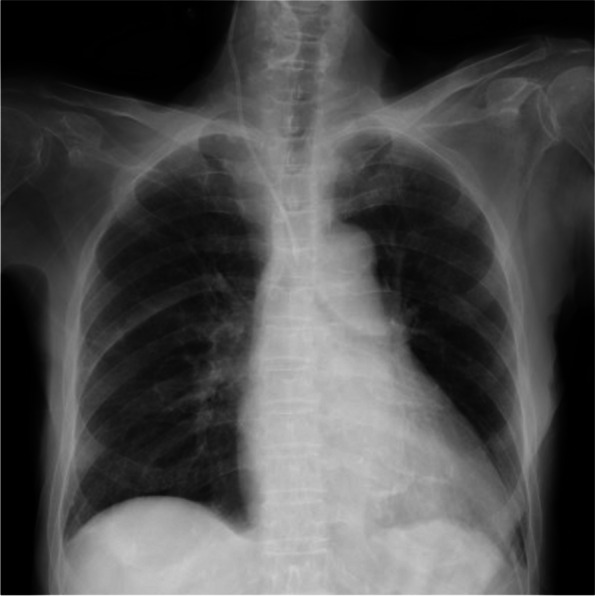
Postprocedural chest roentgenography. The catheter tip appears to overlap the shadow of the trachea where the right brachiocephalic vein or aortic arch exists

An anesthesiologist was urgently called to obtain central venous access. Real-time ultrasound-guided puncture was performed using a cannula-over-needle (18G, needle length 6.35 cm, ARROW® CVC double-lumen catheter, Teleflex, NC, USA) at the midpoint of Sedillot’s triangle. The procedure was then started using the short-axis out-of-plane approach (Sonosite Edge®, Fujifilm Sonosite, WA, USA). Backflow of blood was clearly recognized and looked non-pulsatile. The cannula was connected by an extension tube to saline bottle, and then some drips could be observed. A guidewire was inserted through the cannula, which made it visible in the right internal jugular vein via the short-axis ultrasound view. The anesthesiologist also attempted to confirm the guidewire position via the long-axis view, but it was prevented by the clavicle. Postprocedural chest roentgenography showed that the tip of the catheter was present in the midline (Fig. 1). The anesthesiologist suspected that the catheter had been accidentally misplaced out of the vein. The anesthesiologist inserted a peripheral venous line into the right median cubital vein, and then, transfusion started. After adequate blood transfusion, blood was drawn from the central venous catheter for blood gas analysis under oxygen administration through a nasal prong. The oxygen concentration in the blood gas was 102 mmHg.

**Fig. 2:**
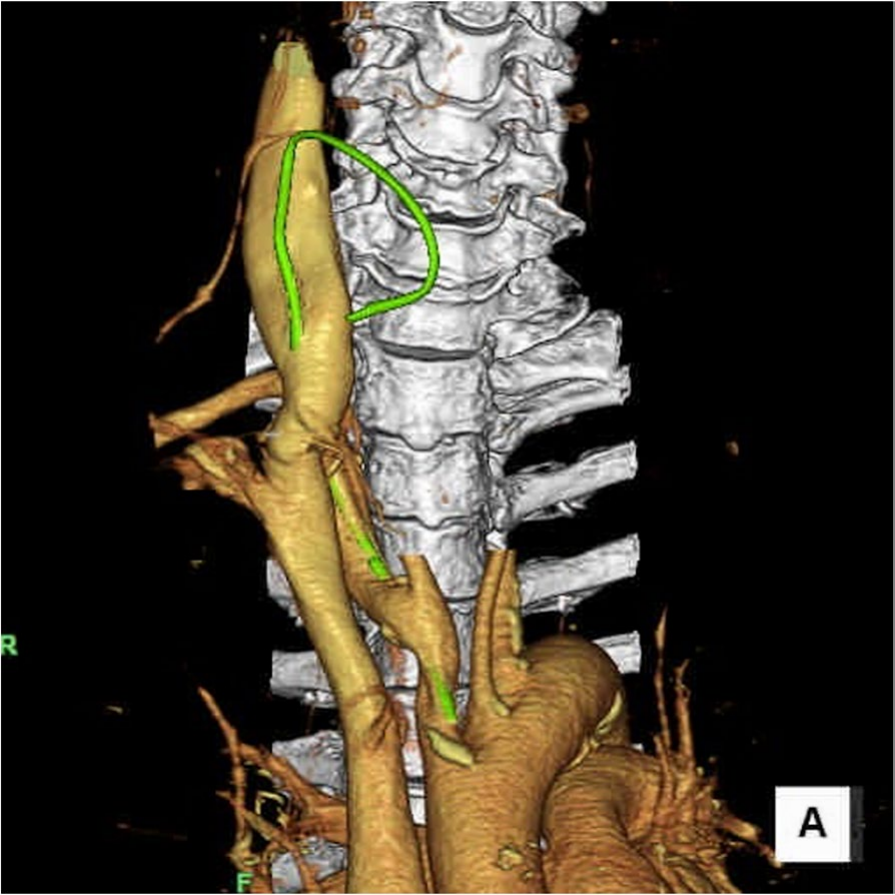
Catheter location with three-dimensional angiography. The catheter (green color) is inserted into the right subclavian artery through the right internal jugular vein. The tip of the catheter is inserted into the right brachiocephalic artery

Computed tomography (CT) and magnetic resonance imaging (MRI) revealed that the catheter had been inserted into the right subclavian artery through the right internal jugular vein (Fig. 2). Open surgery for closure of the subclavian artery perforation was expected to pose a high risk of bleeding. On the other hand, endovascular treatment may have caused occlusion of the right vertebral artery. Contrast-enhanced MRI revealed left-dominant blood supply. The neurosurgeon determined that the right vertebral artery had little blood flow, and the patient’s brain function would not be affected by the occlusion procedure.

**Fig. 3:**
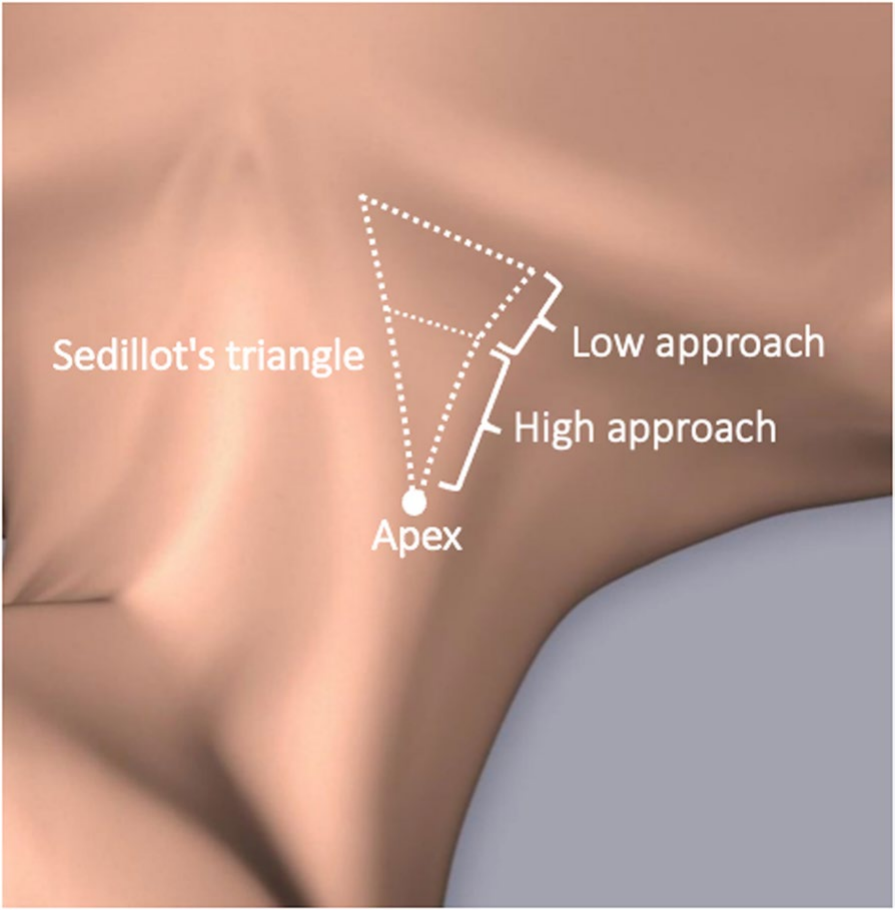
Classification by needle-sticking site for Sedillot’s triangle. In the classification of anatomic landmark techniques by needle-sticking site of Sedillot’s triangle (white dashed triangle), there are two types: high approach (H) and low approach (L). The preferred sticking site in the high approach is the apex (white circle) of the Sedillot’s triangle

A bladder catheter was placed, and continuous bladder lavage was initiated. The next day, the hematuria gradually decreased, and the patient was administered heparin intravenously.

Endovascular treatment was selected by radiologists to close an orifice of the defect caused by the inadvertent insertion of central catheter. Four days later, the patient underwent catheter removal and stent graft insertion into the right subclavian artery (Viabahn VBX 13 × 5 mm W. L. Gore & Associates, Flagstaff, AZ, USA) via right axillary artery access. The catheter was successfully removed, and the orifice of the right vertebral artery needed to be occluded with a stent graft. The patient did not exhibit symptoms of cerebral ischemia after the procedure.

## Discussion

Low circulating blood volume decreases venous pressure [3], and the use of a large bore needle may pose a risk to posterior vein wall penetration [4]. Blaivas et al. reported that the cause of mechanical complication during US-IJV may be related to double-wall penetration [5]. The needle advanced behind this anatomic structure may damage the artery and/or lung. Some guidelines recommend checking the guidewire position before inserting a dilator to prevent subsequent lethal complications [3, 6, 7]. The guidewire position should be confirmed in both the short- and long-axis views [7, 8]. However, in the present case, the guidewire position was confirmed only in the short-axis view. Therefore, the guidewire penetrating the internal jugular vein could not be confirmed. Wakabayashi et al. reported that in an US-IJV, the starting sticking point should be at least 5-cm cephalad from the clavicle to confirm guidewire position in the long-axis ultrasound view [8]. In adult men, the apex of Sedillot’s triangle lies approximately 5-cm cephalad from the clavicle. Therefore, if puncture is started at the apex of Sedillot’s triangle, the guidewire position can be confirmed in the ultrasound long-axis view. However, in the present case, the starting needle-sticking site was at the midpoint of the Sedillot’s triangle, which was insufficient to confirm the guidewire position in the long-axis view. Starting the puncture from the apex of Sedillot’s triangle is called the “high approach,” whereas puncturing from caudal to the midpoint of Sedillot’s triangle is called the “low approach” [9] (Fig. 3). The present case suggests that the high approach is more advantageous than the low approach in that the guidewire position can be reliably confirmed. In the case of a patient with short neck, the use of short-length needle (32–38 mm) for internal jugular venous catheterization is recommended. This needle length is sufficient to puncture the internal jugular vein, and the needle may not reach the blind zone behind the clavicle by using the high approach.

The selection of the optimal needle-sticking site for the anatomic landmark technique has been discussed along with its success rate. It has been shown that US-IJV can be performed in any neck site if the internal jugular vein can be identified. Troianos et al. recommended that the internal jugular venous puncture should be performed in a direction that does not overlap with the common carotid artery to avoid accidental arterial injury [10]. However, arterial injury during US-IJV occurs in the common carotid and subclavian arteries [3, 11]. Therefore, selecting techniques that do not cause double-wall penetration is more important than selecting the optimal needle-sticking site for US-IJV. The authors focused on the sternocleidomastoid muscle, which was used as an anatomic landmark for traditional landmark technique. However, for performing US-IJV, the sternocleidomastoid muscle is not a landmark but an obstacle. This can be attributed to the fact that the thick muscle belly of the sternocleidomastoid muscle is considered a risky needle-sticking site that can interfere with puncture and dilator insertion, which may cause kinking of the guidewire. The high approach recommended in anatomic landmark technique may also be one of the best needle-sticking sites for US-IJV, as it confirms the guidewire position using long-axis ultrasound view and allows easy handling of the needle and smooth insertion of the dilator in Sedillot’s triangle.

The methods used to detect occasional arterial puncture include pressure measurement of the catheter and blood gas analysis [3, 6]. In the present case, the anesthesiologist observed saline dripping through the catheter to confirm the catheter tip location. However, in patients with shock, hypotension, or in whom the cannula tip is in contact with the vein wall, the arterial pressure should be low enough to allow easy spontaneous dripping. Pressure monitoring may also be less reliable in patients with low blood pressure.

**Fig. 4:**
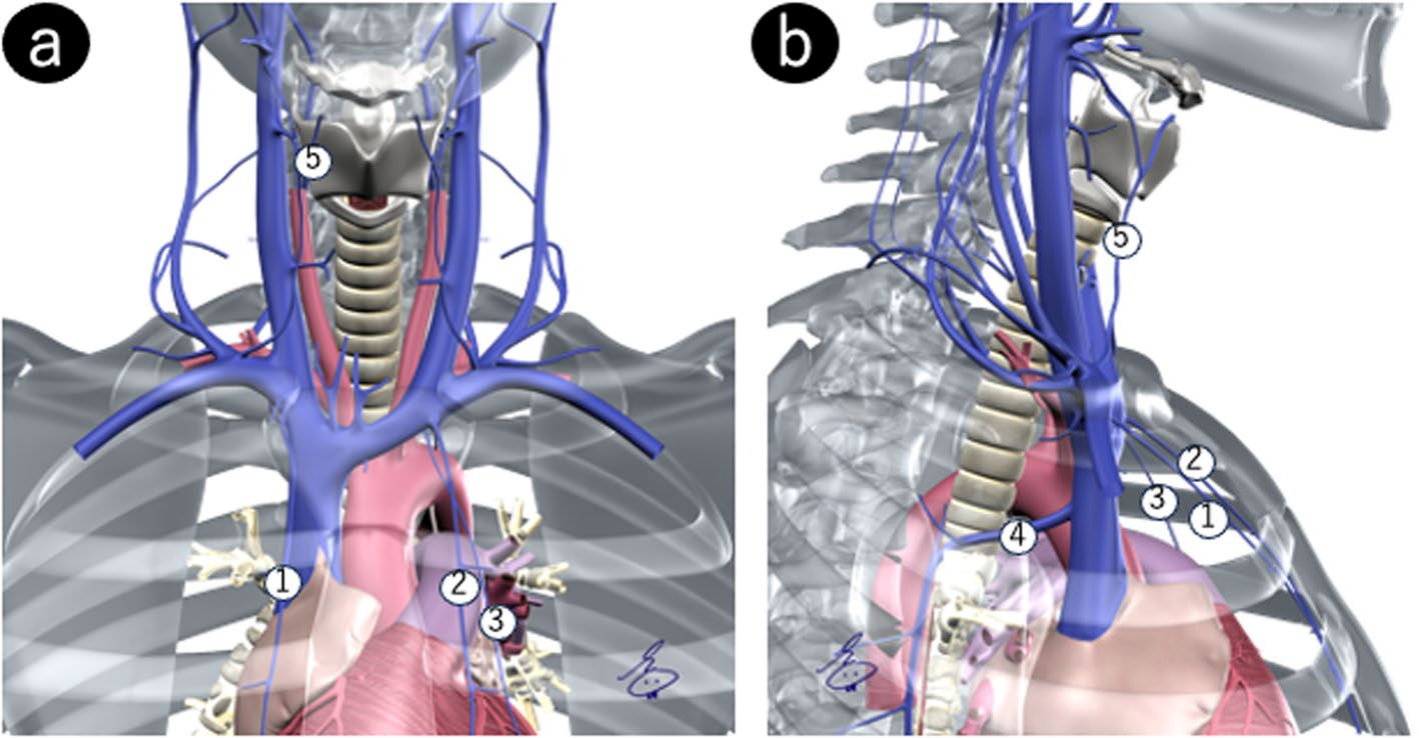
Schematic representation of cervical and thoracic veins for possible misplacement of central venous catheters. **a** Frontal view. **b** Lateral view showing ① right internal thoracic vein, ② left internal thoracic vein, ③ pericardiophrenic vein, ④ azygos vein, ⑤ right inferior thyroid vein

After central venous catheter placement, the location of the catheter is usually confirmed by chest radiography. The inappropriate catheter placements include misplacement into a small vein and extra-venous misplacement. Essentially, the radiographic film should be read on vascular anatomy. Figure 4 shows the small veins tending catheter misplacement. When reading chest radiographs, it is crucial to consider these locations to determine if the catheter was simply misplaced in a small vein or outside a vein. Once it is determined that the catheter is placed at an inappropriate location, an immediate CT or MRI is recommended to determine its exact location and select the befitting treatment for misplacement of a central venous catheter [12].

We experienced a case of central venous catheter misplacement into the right subclavian artery during ultrasound-guided right internal jugular venous catheterization. We recommend the use of the high approach for internal jugular venous catheterization, which allows confirmation of the guidewire position.

## Data Availability

Not applicable.

## Abbreviations

US-IJV: Ultrasound-guided internal jugular venous catheterization
MRI: Magnetic resonance imaging
CT: Computed tomography
